# Genome-Wide Characterization of the LRX Gene Family in Three Cotton Species and Preliminary Evidence Linking *GhLRX2/GhLRX19* to Plant Height in Upland Cotton

**DOI:** 10.3390/ijms27209119

**Published:** 2026-10-14

**Authors:** Jie Li, Xiaohu Ma, Shihua Si, Tianyu Weng, Shuaishuai Chen, Xinyu Zhang, Shen Ren, Feng Liu, Yanjun Li, Fei Xue, Lizhong Xuan, Jie Sun

**Affiliations:** 1 College of Agriculture, Shihezi University, Shihezi 832000, China; li_jie@stu.shzu.edu.cn (J.L.); maxiaohu@stu.shzu.edu.cn (X.M.); sishihua@stu.shzu.edu.cn (S.S.); wengtianyu@stu.shzu.edu.cn (T.W.); cheng_s_s@163.com (S.C.); zhxy@shzu.edu.cn (X.Z.); renshen@stu.shzu.edu.cn (S.R.); liufeng@shzu.edu.cn (F.L.); liyanjun@shzu.edu.cn (Y.L.); xuefei@shzu.edu.cn (F.X.); 2 Agricultural Technology Extension Station, Xinjiang Production and Construction Corps, Ürümqi 830011, China

**Keywords:** *Gossypium hirsutum*, leucine-rich repeat extensin, genome-wide identification, plant height, virus-induced gene silencing

## Abstract

Plant height is an important agronomic trait affecting cotton architecture and mechanized production. Leucine-rich repeat extensins (LRXs) are cell-wall-associated proteins involved in cell-wall organization, cell elongation, and cell-wall integrity signaling. In this study, genome-wide analysis of *Gossypium hirsutum*, *G. arboreum*, and *G. raimondii* identified 74 LRX loci, comprising 36 GhLRX, 19 GaLRX, and 19 GrLRX genes. These 74 loci were represented by 79 annotated protein models, including five additional models from three GrLRX loci. Maximum-likelihood phylogenetic analysis of 74 representative cotton proteins and 11 *Arabidopsis *proteins assigned them to four descriptive groups. Of the 79 protein models, 75 were predicted to contain Sec/SPI-type signal peptides. Conserved motif and gene structure analyses revealed broadly similar organization with variation among members and alternative isoforms. Promoter analysis identified putative hormone- and stress-related cis-acting elements. Collinearity analyses revealed syntenic relationships within upland cotton and with its diploid relatives. Public transcriptome data showed variation in GhLRX transcript abundance across tissues, developmental stages, and abiotic stress conditions. The homoeologous genes *GhLRX2* and *GhLRX19* showed relatively high transcript abundance in stems and 20-day-post-anthesis fibers and were selected for preliminary investigation. Virus-induced gene silencing (VIGS) treatment targeting a sequence shared by *GhLRX2* and *GhLRX19* was associated with lower combined transcript abundance and reduced plant height. These findings provide preliminary evidence linking this shared-sequence VIGS treatment to reduced plant height, while the individual responses and functions of the two homoeologs remain unresolved.

## 1. Introduction

Cotton is one of the most important fiber crops worldwide. Cotton fiber is an important natural raw material for the textile industry, and its yield and quality are important determinants of the competitiveness of the cotton textile sector [1,2]. Plant height is a key component of cotton plant architecture and affects canopy structure and light interception. It is also closely associated with planting-density adaptation, canopy ventilation and light penetration, mechanized management, and yield formation [3,4,5]. Appropriate plant height, together with compact branching architecture, can facilitate high-density planting and mechanical harvesting [6]. Excessive plant height increases the risk of lodging, whereas insufficient plant height may restrict vegetative growth and the formation of effective fruiting branches [7,8,9,10]. Cotton plant height is mainly determined by the number of stem nodes and internode length, with internode elongation being regulated by cell division, cell expansion, phytohormones, and environmental factors [11,12]. Although quantitative trait loci (QTLs), hormone-regulatory factors, and transcription factors associated with cotton plant height have been reported [6,9,11,13,14,15], the molecular mechanisms linking cell wall status, cell elongation, and plant height formation remain to be further elucidated.

Plant cell walls provide an essential structural basis for maintaining cell shape, mechanical strength, and organ development. Primary cell walls are mainly composed of cellulose, hemicellulose, pectic polysaccharides, and small amounts of structural proteins. Their dynamic assembly and remodeling directly affect cell wall plasticity and enable directional cell elongation driven by turgor pressure [16,17,18]. In addition to providing mechanical support, the cell wall participates in cell wall integrity signaling. Plants perceive changes in cell wall mechanical properties and structure through cell wall-associated receptor kinases, ion channels, and extracellular signaling molecules, thereby regulating cell wall remodeling, cell elongation, and stress adaptation [19,20,21].

Extensins (EXTs) are hydroxyproline-rich cell wall glycoproteins. They typically contain extensin repeat motifs and contribute to cell wall network formation through glycosylation and intermolecular cross-linking. Leucine-rich repeat extensin (LRX) proteins are chimeric members of the extensin superfamily. Typical LRX proteins consist of an N-terminal leucine-rich repeat (LRR) domain and a C-terminal extensin-like domain [22,23,24]. The N-terminal LRR region primarily mediates protein–protein interactions and can bind RAPID ALKALINIZATION FACTOR (RALF) peptides or other cell wall-associated components [25,26,27]. The C-terminal extensin-like region is enriched in hydroxyproline and associated repeat sequences and may contribute to cell wall network formation and stabilization through glycosylation and tyrosine-mediated cross-linking [28,29].

Previous studies have demonstrated that LRX proteins are involved in cell elongation, cell wall integrity maintenance, and reproductive development. In *Arabidopsis thaliana*, the *lrx1* single mutant exhibits shortened and morphologically abnormal root hairs, and the *lrx1 lrx2* double mutant displays more severe root hair rupture phenotypes, indicating a synergistic interaction between *LRX1* and *LRX2* in root hair cell wall formation [22]. The *lrx3 lrx4 lrx5* triple mutant exhibits severe root growth inhibition accompanied by changes in pectin and cellulose composition in the cell wall [30]. In addition, mutations in LRX genes cause severe defects in pollen germination and pollen tube growth [31,32]. RALF signaling peptides regulating plant reproductive development have been confirmed to bind to the LRR domains of LRX proteins and modulate pollen tube development [26], further supporting a role of LRX proteins in reproductive development.

Both stem elongation and cotton fiber development are closely associated with the dynamic assembly and remodeling of the cell wall. Therefore, we hypothesized that LRX family members may be involved in cotton plant height formation and fiber development. Previous studies have identified and characterized LRX family members in upland cotton (*Gossypium hirsutum* L.), Asian cotton (*G. arboreum*), and *G. raimondii*, primarily to investigate their potential roles in cotton disease resistance, particularly resistance to *Verticillium* wilt [33]. However, whether and how LRX family members participate in cotton plant height formation and fiber development remains unclear. In this study, the LRX family was comparatively analyzed in upland cotton, *G. arboreum*, and *G. raimondii*. The physicochemical properties of the encoded proteins, phylogenetic relationships, gene structures, and other relevant characteristics were systematically examined, with particular emphasis on their potential association with plant height development. Based on publicly available transcriptome expression profiles, *GhLRX2* and *GhLRX19*, a pair of A- and D-subgenome homoeologs with relatively high transcript abundance in stems and fibers at 20 days post-anthesis (DPA), were selected for preliminary investigation. Virus-induced gene silencing (VIGS) targeting a sequence shared by *GhLRX2* and *GhLRX19* was used to examine the potential association of this homoeologous gene pair with plant height. Longitudinal stem sections were also examined to provide descriptive observations of cortical parenchyma cell morphology. This approach assessed the phenotype associated with targeting a sequence shared by the homoeologous pair rather than resolving the individual functions of the two genes. This study provides a basis for further investigation of cotton LRX genes and preliminary evidence linking *GhLRX2*/*GhLRX19* to plant height in upland cotton.

## 2. Results

### 2.1. Identification and Basic Characteristics of LRX Family Members in Three Cotton Species

To systematically characterize the LRX gene family in cotton, we conducted a genome-wide survey and identified 74 LRX loci, including 36 GhLRX loci in *Gossypium hirsutum*, 19 GaLRX loci in *G. arboreum*, and 19 GrLRX loci in *G. raimondii*. Based on their physical locations on the chromosomes, the loci were sequentially designated *GhLRX1*–*GhLRX36*, *GaLRX1*–*GaLRX19*, and *GrLRX1*–*GrLRX19*, respectively. Annotated alternative models from the same locus were distinguished by decimal suffixes, namely *GrLRX1.1*–*GrLRX1.4*, *GrLRX11.1*–*GrLRX11.2*, and *GrLRX14.1*–*GrLRX14.2*. Thus, the 74 loci were represented by 79 predicted protein models.

The physicochemical properties of the identified LRX proteins were analyzed using the ExPASy ProtParam tool. The genomic spans of the 74 LRX loci ranged from 770 to 6042 bp, whereas the 79 predicted protein sequences ranged from 231 to 762 amino acids in length. The physicochemical properties of GhLRX35 were recorded as NA and excluded from the corresponding statistical summaries because its annotated protein sequence contained an extended region of undetermined amino acid residues (X) (Table S1). The predicted molecular weights of the proteins ranged from 25.51 to 81.93 kDa, and their theoretical isoelectric points (pI) ranged from 4.63 to 9.85. Among the 78 protein sequences, 43 had theoretical pI values above 7.0, accounting for 55.1% of all analyzed LRX protein sequences. The Instability Index, Aliphatic Index, and GRAVY values ranged from 30.14 to 103.49, 51.06 to 102.30, and −0.841 to 0.244, respectively. Based on their GRAVY values, 55 proteins were predicted to be hydrophilic, whereas 23 were predicted to be hydrophobic (Table S1). These results indicate that the LRX proteins in the three cotton species possess diverse physicochemical properties.

To further investigate the potential secretory characteristics of LRX proteins, N-terminal signal peptides were predicted using SignalP 6.0 with the organism group set to Eukarya. In total, 79 predicted protein sequences representing 74 LRX loci were analyzed, including additional models from *GrLRX1*, *GrLRX11*, and *GrLRX14*. Among these sequences, 75 (94.9%) were predicted to contain Sec/SPI-type signal peptides, including 32 of the 36 GhLRX sequences, all 19 GaLRX sequences, and all 24 GrLRX sequences. The alternative protein models corresponding to each of these three GrLRX loci showed identical signal-peptide predictions.

Among the 75 proteins classified as SP, 73 had SP(Sec/SPI) class probabilities above 0.90. The two proteins with lower probabilities were GaLRX17 (0.853992) and GrLRX8 (0.833166), although both were classified as SP by SignalP 6.0. The predicted cleavage sites ranged from positions 12–13 to 37–38, with cleavage-site probabilities ranging from 0.3876 to 0.9835. These cleavage-site probabilities refer to the predicted cleavage positions and are distinct from the SP(Sec/SPI) class probabilities. No Sec/SPI-type signal peptide was predicted for GhLRX9, GhLRX15, GhLRX20, or GhLRX36. Overall, these predictions suggest that most analyzed LRX proteins may enter the classical secretory pathway, consistent with potential extracellular or cell-wall localization (Table S2).

### 2.2. Phylogenetic Analysis of the LRX Family in Three Cotton Species

**Figure 1:**
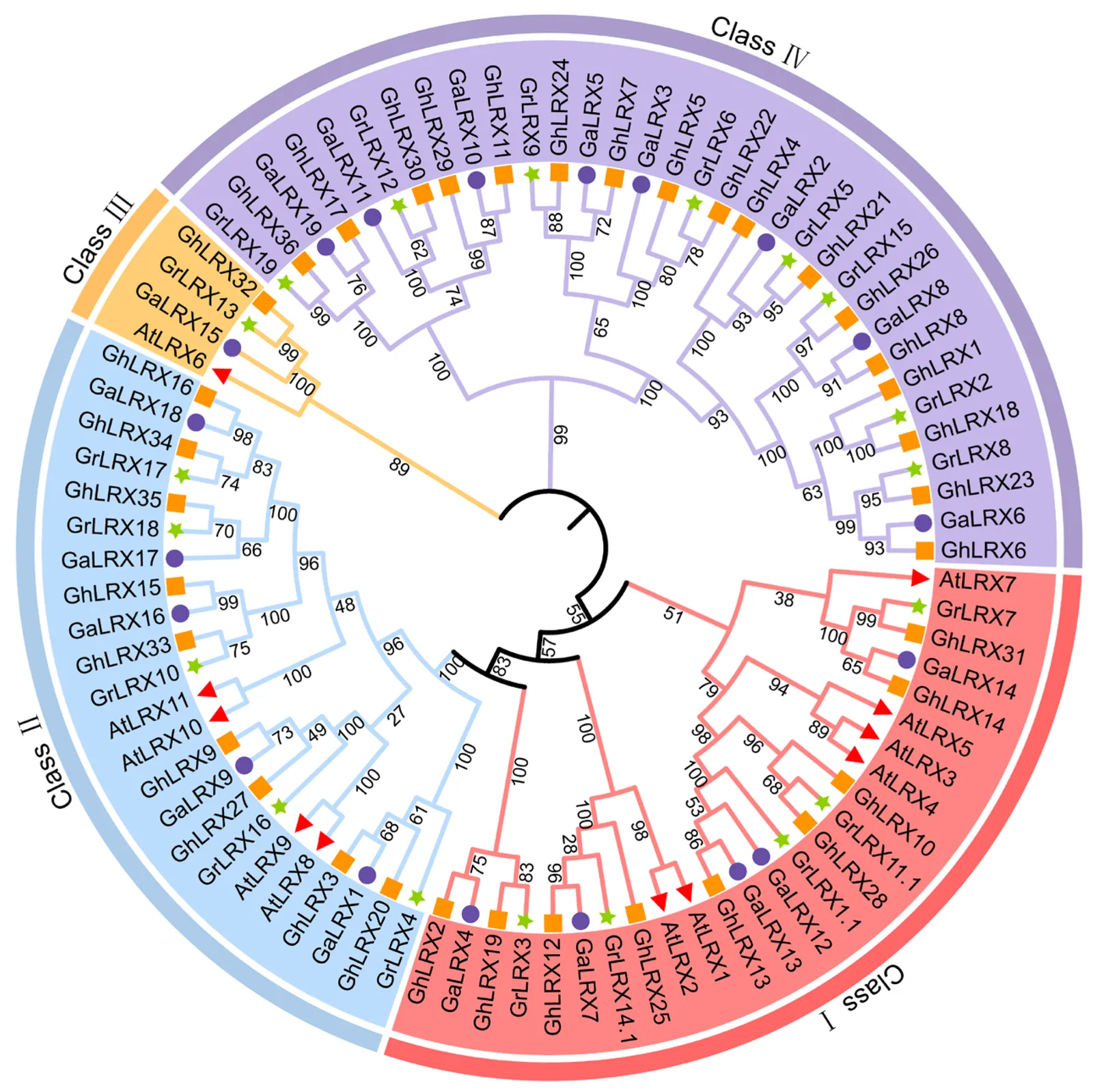
Unrooted maximum-likelihood phylogenetic tree of LRX proteins from *Gossypium hirsutum*, *G. arboreum*, *G. raimondii*, and *Arabidopsis thaliana*. The analysis included 85 protein sequences, comprising 74 representative cotton LRX sequences (36 GhLRX, 19 GaLRX, and 19 GrLRX) and 11 AtLRX sequences. One representative protein sequence per cotton LRX locus was included, and decimal suffixes in tip labels identify the selected annotated models. The four designated groups (Class I–IV) are highlighted in red, blue, yellow, and purple, respectively. The black branches in the center represent internal connections among the four color-coded classes. Orange squares, purple circles, green stars, and red triangles indicate proteins from *G. hirsutum*, *G. arboreum*, *G. raimondii*, and *A. thaliana*, respectively. Numbers at internal nodes indicate bootstrap support values (%).

To investigate the phylogenetic relationships of the LRX family, a maximum-likelihood phylogenetic tree was constructed using 85 protein sequences, comprising 74 representative cotton LRX protein sequences and 11 AtLRX protein sequences. One representative protein sequence was included for each cotton LRX locus, yielding 36 GhLRX, 19 GaLRX, and 19 GrLRX sequences. Based on the phylogenetic topology and with reference to the *Arabidopsis* LRX members, the analyzed proteins were assigned to four groups, designated Class I–IV. Class I contained 25 proteins, including AtLRX1–5 and AtLRX7, whereas Class II contained 23 proteins, including AtLRX8–11. Class III comprised GhLRX32, GaLRX15, GrLRX13, and AtLRX6. Class IV contained 33 cotton LRX proteins and no *Arabidopsis* LRX members (Figure 1). To evaluate the potential influence of the undetermined amino acid region in GhLRX35 on its phylogenetic placement, we reconstructed the phylogenetic tree after removing this region. GhLRX35 retained the same phylogenetic position as in the original tree, indicating that the uncertain region did not alter its inferred placement.

### 2.3. Conserved Motif and Gene Structure Analyses of the LRX Family in Three Cotton Species

**Figure 2:**
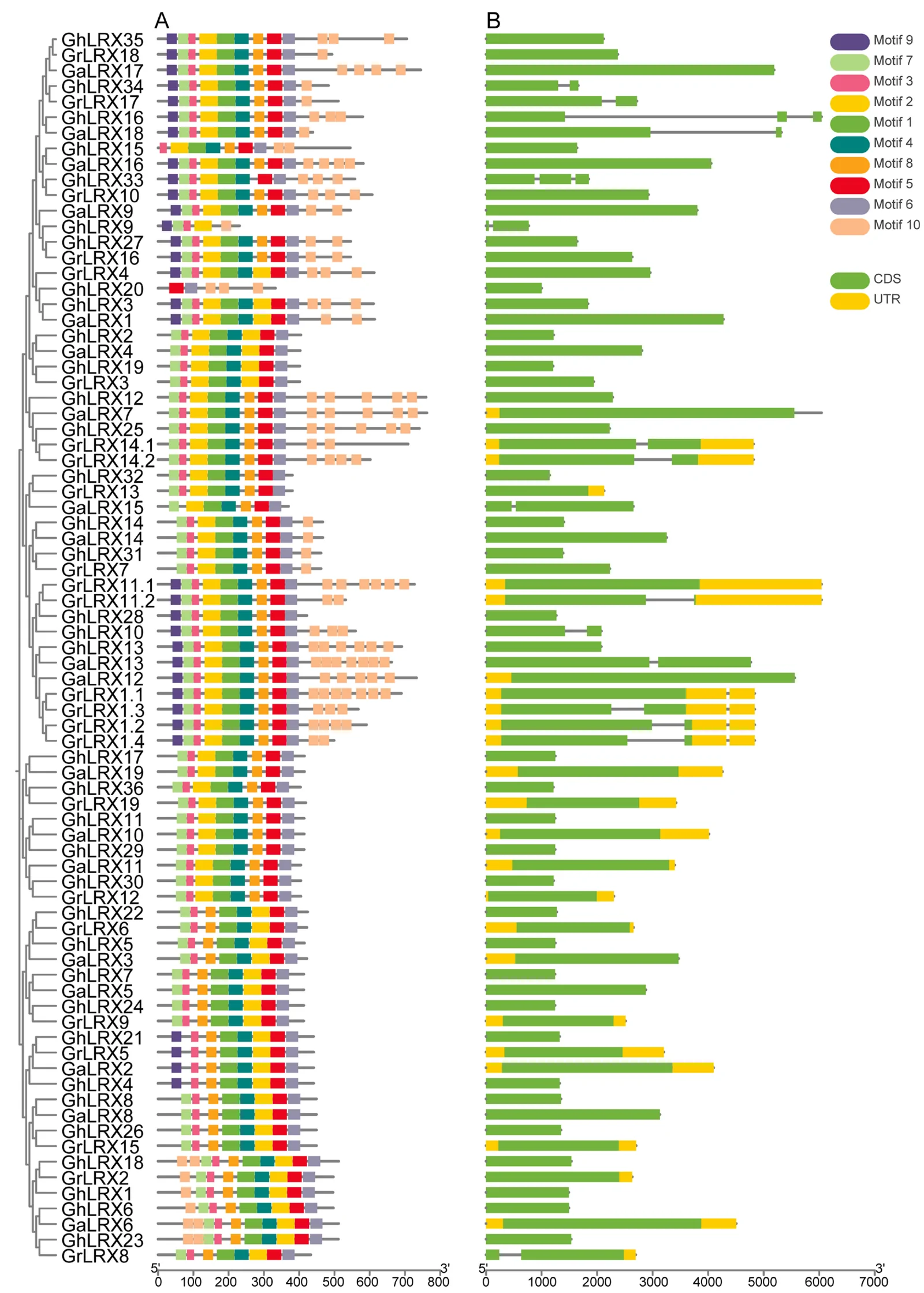
Conserved motif and exon–intron structure analyses of the LRX family in three cotton species. (**A**) Distribution of conserved motifs identified using MEME among 79 predicted LRX protein sequences, comprising 36 GhLRX, 19 GaLRX, and 24 GrLRX sequences representing 36, 19, and 19 loci, respectively. Different-colored boxes represent the ten identified motif types. (**B**) Exon–intron structures of the corresponding 79 annotated transcript models from 74 LRX loci, based on the genome annotations of the three cotton species. Alternative models from three *GrLRX* loci are designated GrLRX1.1–GrLRX1.4, GrLRX11.1–GrLRX11.2, and GrLRX14.1–GrLRX14.2. Model labels refer to predicted proteins in panel (**A**) and their corresponding transcript models in panel (**B**). Green boxes indicate coding sequences (CDSs), yellow boxes indicate untranslated regions (UTRs), and gray lines connecting exons indicate introns. The scales in panels (**A**) and (**B**) indicate amino acid and nucleotide positions, respectively.

MEME analysis identified 10 conserved motif types among 79 predicted LRX protein sequences representing 74 loci from the three cotton species. LRX proteins belonging to the same phylogenetic group generally exhibited similar motif compositions and arrangements, suggesting relatively conserved motif organization within these groups. Individual proteins contained three to ten motif types, and 26 proteins contained all ten identified types. Most LRX proteins shared a relatively conserved core motif arrangement, whereas several members differed in motif presence, copy number, or distribution. In particular, Motif 10 occurred repeatedly in the C-terminal regions of some proteins, with variation in the number and positions of these repeats. Alternative predicted protein models annotated at the *GrLRX1*, *GrLRX11*, and *GrLRX14* loci retained the same motif types within each locus but differed in the copy number and distribution of Motif 10. These observations revealed variation in conserved motif organization among LRX family members and among alternative predicted protein models from the same locus (Figure 2A).

Gene structure analysis revealed that the 79 annotated transcript models from 74 LRX loci generally had relatively simple exon–intron organizations, with few or no introns. Among these transcript models, 17 contained at least one intron, whereas the remaining 62 were intronless, indicating that intronless structures predominated among the analyzed transcript models. In addition, annotated alternative transcript models from the *GrLRX1*, *GrLRX11*, and *GrLRX14* loci exhibited differences in their exon–intron structures. For example, *GrLRX11.1* was intronless, whereas *GrLRX11.2* contained an intron. These differences illustrate variation among the annotated transcript structures within the LRX family. Nevertheless, the exon–intron structures of most analyzed LRX transcript models remained relatively simple (Figure 2B).

### 2.4. Cis-Acting Element Analysis of LRX Gene Promoters in Three Cotton Species

**Figure 3:**
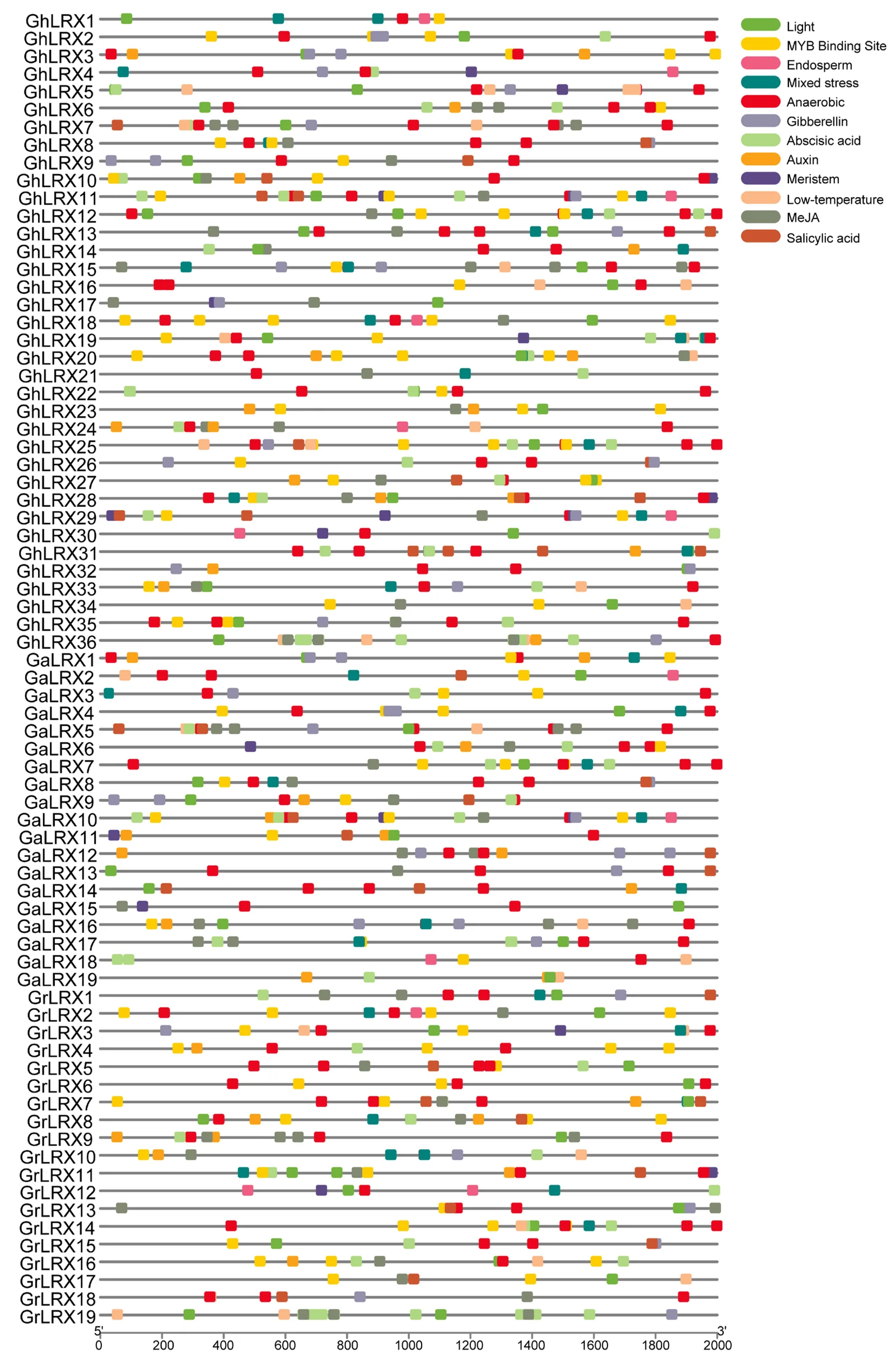
Distribution of predicted cis-acting elements in the promoter regions of 74 LRX loci from three cotton species. One promoter sequence was analyzed per locus. Promoter regions were defined as the 2000-bp sequences upstream of the translation initiation sites of the representative transcript models. Putative cis-acting elements were predicted using PlantCARE and visualized with TBtools-II v2.487. Labels indicate LRX loci, and different colors indicate different types of putative cis-acting elements.

To analyze the potential cis-regulatory features of the LRX gene family, 74 promoter sequences, one per LRX locus from the three cotton species, were analyzed using cis-acting element prediction. The analysis predicted multiple cis-acting elements associated with phytohormone and environmental stress responses in these promoter sequences. Among the hormone-responsive elements, MeJA-responsive elements were the most widely distributed, being predicted in 48 promoter sequences, followed by ABA-responsive elements and gibberellin-responsive elements, which were predicted in 46 and 36 promoter sequences, respectively. Salicylic acid (SA)-responsive elements and auxin-responsive elements were predicted in 30 and 29 promoter sequences, respectively, whereas low-temperature-responsive elements were predicted in 24 promoter sequences (Figure 3). The composition and distribution of the predicted cis-acting elements varied among the LRX promoter sequences from the three cotton species. These findings provide descriptive information on putative regulatory elements and identify potential directions for investigating hormone- and stress-related regulation of LRX genes, but do not establish functional responses to these signals.

### 2.5. Chromosomal Localization and Collinearity Analysis of the GhLRX Gene Family

**Figure 4:**
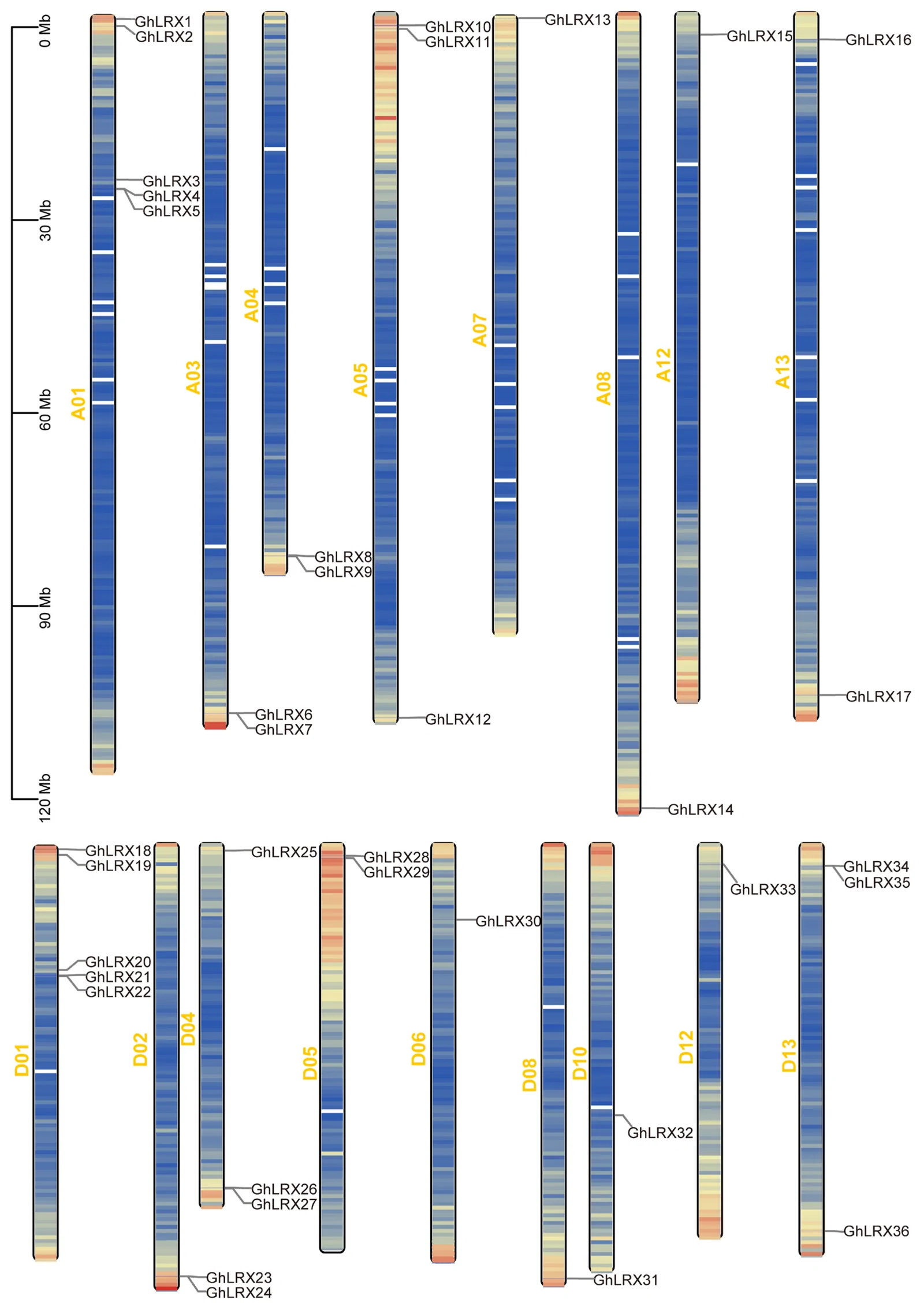
Chromosomal distribution of *GhLRX* genes in upland cotton. The *GhLRX* genes are distributed across 17 chromosomes of the At and Dt subgenomes. The scale bar represents 120 Mb.

Chromosomal localization analysis revealed that the 36 GhLRX genes were unevenly distributed across 17 chromosomes of upland cotton. Of these, 17 genes were located in the At subgenome, whereas 19 were located in the Dt subgenome, indicating a slight difference in gene number between the two subgenomes (Figure 4). Chromosomes A01 and D01 each contained five GhLRX genes, the highest number observed on any individual chromosome. Chromosomes A05, D04, and D13 each contained three GhLRX genes, whereas chromosomes A03, A04, A13, D02, and D05 each contained two. The remaining seven chromosomes each contained a single GhLRX gene.

**Figure 5:**
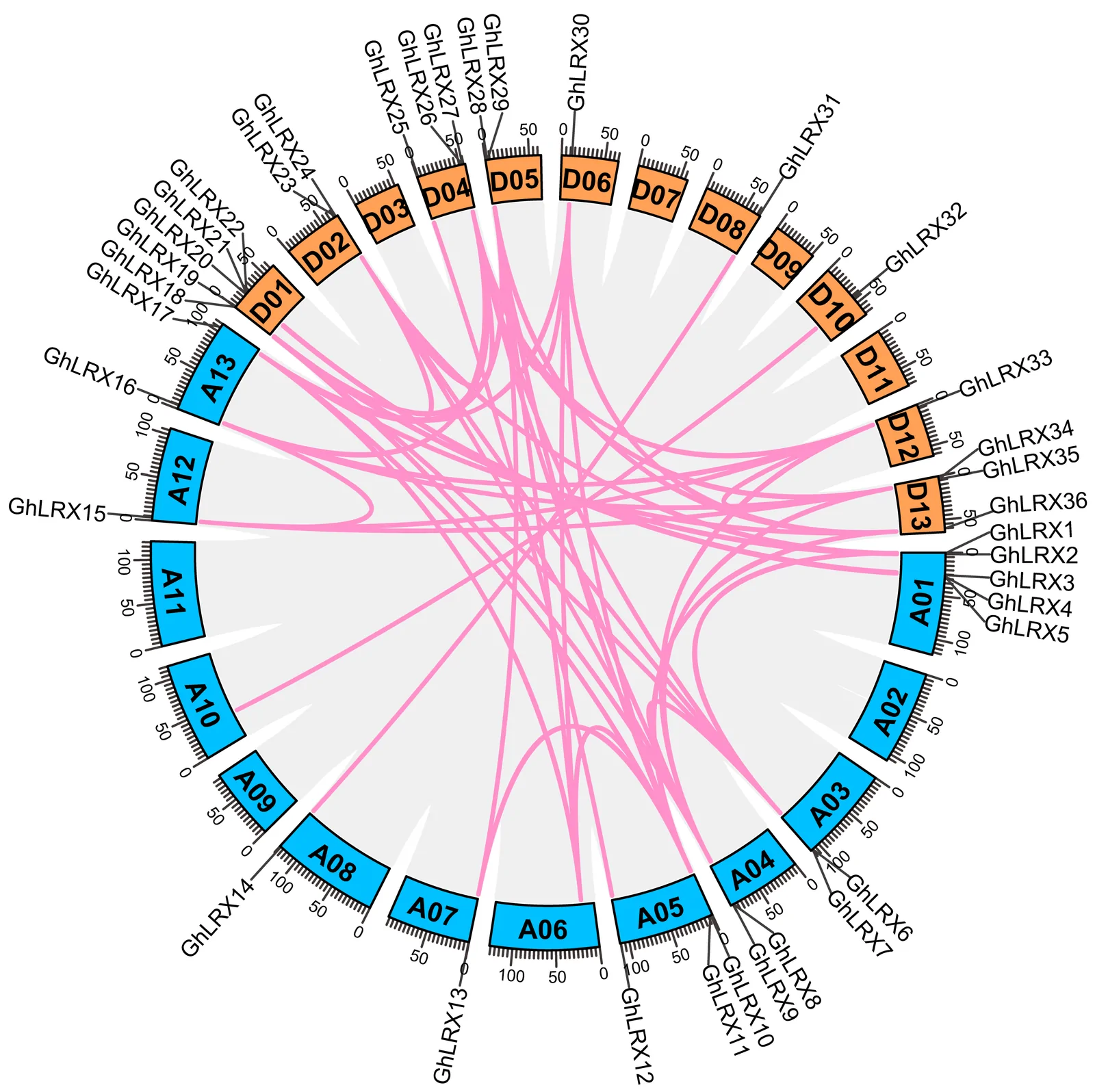
Chromosomal distribution and collinearity relationships of the GhLRX gene family in upland cotton. Blue and orange segments represent chromosomes of the A and D subgenomes, respectively, and the gene names indicate the positions of the corresponding GhLRX genes. Pink ribbons represent collinear relationships identified in the syntenic regions containing the GhLRX genes.

To investigate the expansion and evolutionary history of the GhLRX gene family, an intraspecific collinearity analysis was performed using the MCScanX module implemented in TBtools-II v2.487. A total of 50 collinear gene pairs involving the identified GhLRX loci were detected. Among these, 34 intersubgenomic collinear gene pairs were identified between the At and Dt subgenomes. Of the 50 pairs, 44 connected two identified GhLRX genes, whereas the remaining six involved an identified GhLRX gene and a collinear partner outside the final family dataset. Duplication-mode analysis further showed that 34 of the 36 identified GhLRX genes were classified as WGD/segmental duplicates, whereas *GhLRX20* and *GhLRX35* were classified as dispersed and tandem duplicates, respectively (Figure 5; Table S3). These results indicate that WGD/segmental duplication was the predominant duplication category among the identified GhLRX genes, whereas dispersed and tandem duplicates accounted for smaller proportions of the family.

To assess the selective pressures acting on duplicated genes associated with the GhLRX loci, *Ka/Ks* ratios were calculated for the 50 collinear gene pairs involving identified GhLRX loci. All 50 gene pairs exhibited *Ka/Ks* ratios below 1, indicating that these duplicated gene pairs generally evolved under purifying selection. Moreover, 44 gene pairs had *Ka/Ks* ratios below 0.3, accounting for 88.0% of the analyzed pairs and suggesting relatively strong functional constraints on most of these duplicated gene pairs. The remaining six gene pairs had *Ka/Ks* ratios ranging from 0.358 to 0.723. Although these pairs may have experienced relatively weaker purifying selection, their *Ka/Ks* ratios remained below 1, indicating that they were also subject to purifying selection (Table S4).

### 2.6. Interspecific Collinearity Analysis of the GhLRX Gene Family

**Figure 6:**
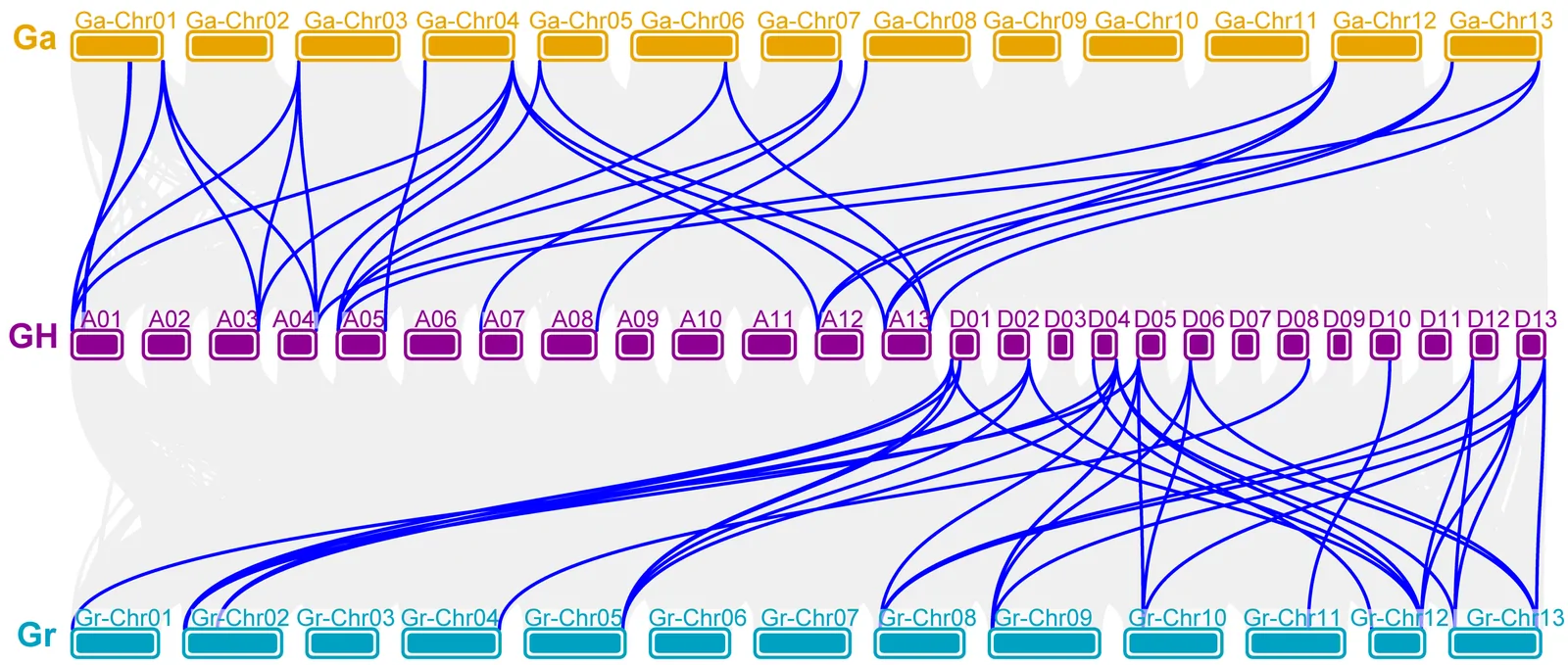
Interspecific collinearity analysis between *Gossypium arboreum* and *Gossypium hirsutum,* and between *Gossypium raimondii* and *Gossypium hirsutum*. Gray lines indicate genome-wide collinear blocks, and blue lines highlight collinear relationships involving the identified GhLRX loci.

To assess the syntenic conservation of the LRX family between *Gossypium hirsutum* and its diploid relatives, the At and Dt subgenomes were compared with *G. arboreum* (Ga) and *G. raimondii* (Gr), respectively. A total of 31 GhAt–Ga and 36 GhDt–Gr collinear gene pairs involving the identified GhLRX genes were detected. Of these, 27 GhAt–Ga and 33 GhDt–Gr pairs connected genes identified as LRX family members in both species. The remaining four GhAt–Ga pairs and three GhDt–Gr pairs involved an identified GhLRX locus and a collinear partner not included in the final LRX dataset of the corresponding diploid species. These LRX–LRX relationships involved 15 of the 17 GhAt LRX genes (88.24%), 15 of the 19 GaLRX genes (78.95%), 15 of the 19 GhDt LRX genes (78.95%), and 15 of the 19 GrLRX genes (78.95%). Several LRX genes exhibited one-to-many or many-to-one collinear relationships. For example, *GhLRX11* and *GhLRX27* were each collinear with three GaLRX and three GrLRX genes, respectively, whereas *GaLRX9* and *GrLRX15* each corresponded to three LRX genes in the GhAt and GhDt subgenomes, respectively. Overall, these results indicate that a substantial proportion of the identified LRX genes retained syntenic relationships between upland cotton and its diploid relatives (Figure 6; Table S11).

### 2.7. Expression Profiles of GhLRX Genes Across Tissues and Developmental Stages

**Figure 7:**
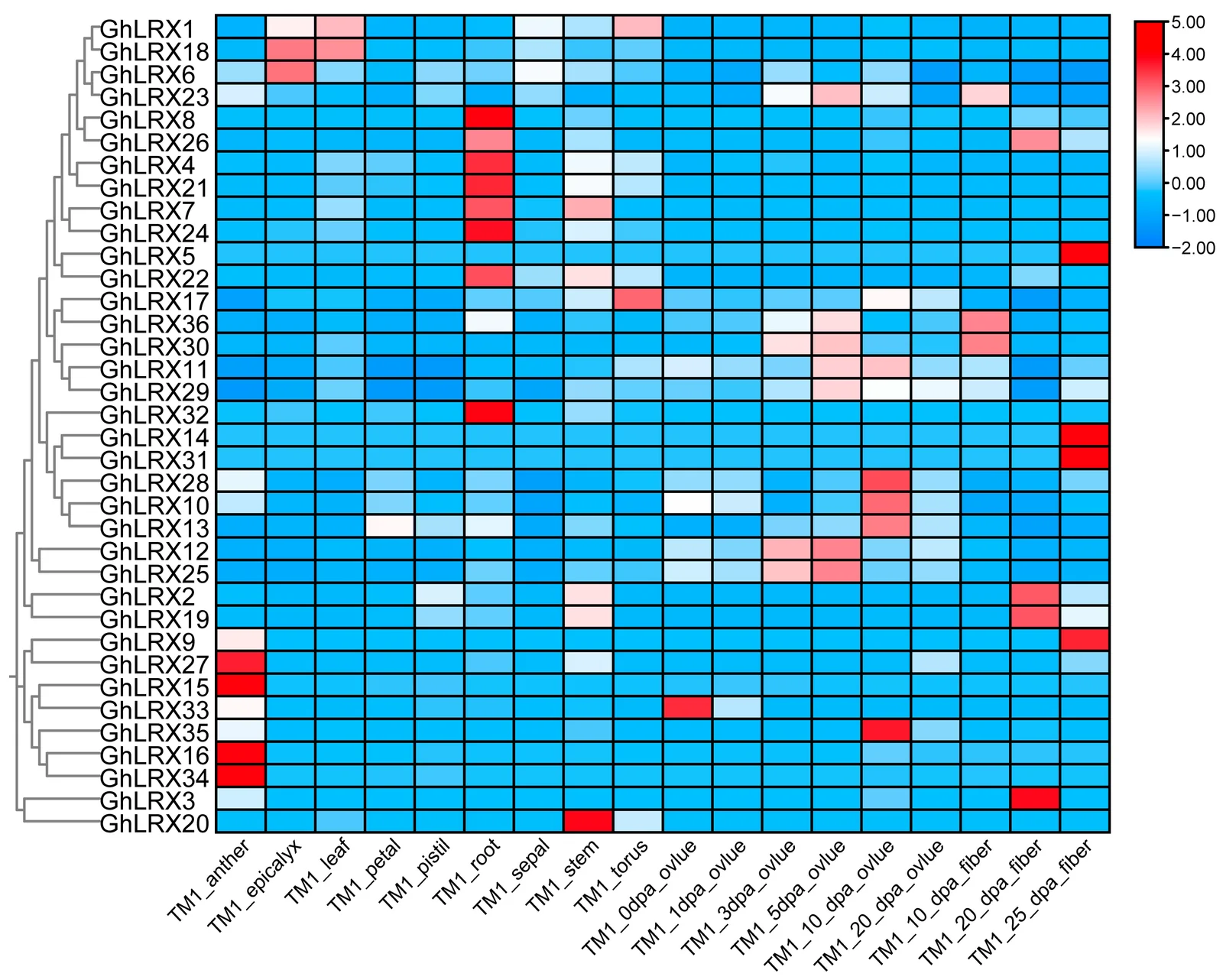
Relative expression profiles of GhLRX genes across different tissues and developmental stages of upland cotton. Expression data were obtained from the Zhejiang University Cotton Database (http://cotton.zju.edu.cn/). The values provided by the database were normalized and averaged across biological replicates and do not represent raw RNA-seq read counts. For heatmap visualization, expression values were row-scaled (Row Scale) in TBtools-II to highlight the relative expression patterns of individual GhLRX genes across different tissues and developmental stages. The color gradient represents row-scaled *Z*-scores, with blue and red indicating relatively low and high values, respectively.

To characterize the expression profiles of GhLRX genes across tissues and developmental stages, publicly available RNA-seq expression data from *Gossypium hirsutum* were analyzed. *GhLRX2* and *GhLRX19* exhibited relatively high transcript abundance in stems and fibers at 20 days post-anthesis (DPA), whereas *GhLRX18* showed relatively high transcript abundance in the epicalyx, leaves, and sepals. *GhLRX10* and *GhLRX28* were broadly expressed across the examined samples, with the highest transcript abundance among the examined samples in 10-DPA ovules. In contrast, *GhLRX12* and *GhLRX25* showed relatively high transcript abundance in 3- and 5-DPA ovules. Several members, including *GhLRX15*, *GhLRX16*, and *GhLRX34*, exhibited higher transcript abundance in anthers than in the other samples examined, suggesting possible anther-preferential expression patterns (Figure 7; Table S7).

### 2.8. Expression Profiling of GhLRX Genes Under Abiotic Stress

**Figure 8:**
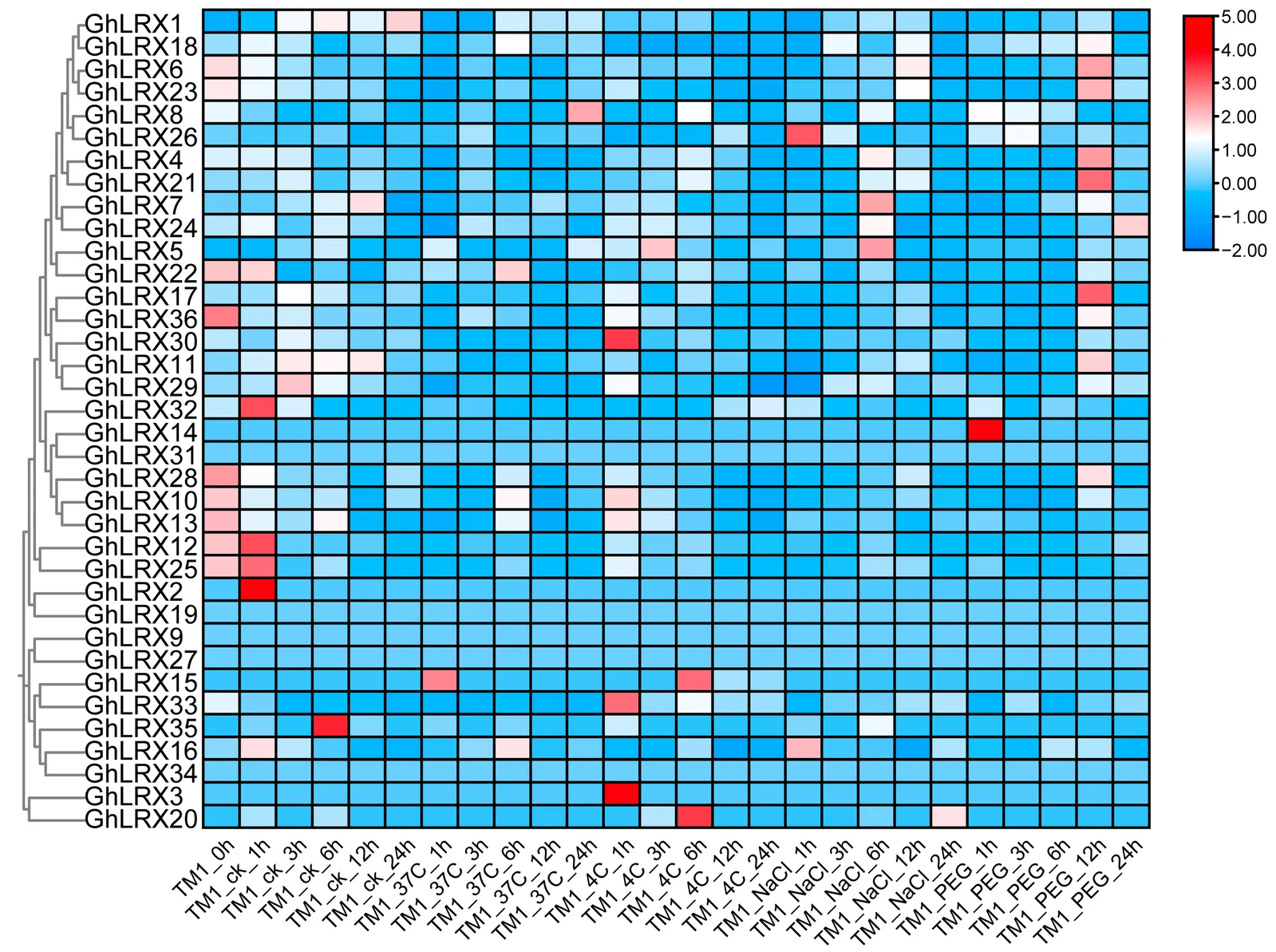
Relative expression profiles of GhLRX genes in upland cotton under abiotic stress treatments. Expression data were obtained from the Zhejiang University Cotton Database (http://cotton.zju.edu.cn/). The values provided by the database were normalized and averaged across biological replicates and do not represent raw RNA-seq read counts. For heatmap visualization, expression values were row-scaled (Row Scale) in TBtools-II to highlight the relative expression patterns of individual GhLRX genes across different stress treatments. The color gradient represents row-scaled *Z*-scores, with blue and red indicating relatively low and high values, respectively.

To examine the expression profiles of GhLRX genes under abiotic stress conditions, publicly available transcriptome data from TM-1 subjected to high-temperature (37 °C), low-temperature (4 °C), NaCl, or PEG treatments were analyzed. Transcript abundance varied among genes, treatments, and sampling times, with many genes showing low or undetectable expression in the examined samples. *GhLRX1* and *GhLRX18* exhibited relatively high transcript abundance across multiple conditions. Compared with the time-matched controls, *GhLRX18* showed higher transcript abundance at 6 h under the 37 °C treatment and at several time points under NaCl and PEG treatments, whereas lower values were observed throughout the 4 °C treatment. *GhLRX10* and *GhLRX28* also exhibited higher transcript abundance than the corresponding controls at 12 h under both NaCl and PEG treatments (Figure 8; Table S8).

### 2.9. Preliminary Investigation of GhLRX2/GhLRX19 by VIGS-Mediated Targeting

Plant height is an important agronomic trait that influences cotton plant architecture, canopy structure, and adaptability to cultivation practices. Its formation is closely associated with processes such as cell division, cell elongation, and cell-wall formation and remodeling. Given the relatively high transcript abundance of the homoeologous genes *GhLRX2* and *GhLRX19* in stems and 20-DPA fibers, these genes were selected for preliminary investigation using virus-induced gene silencing (VIGS). Because *GhLRX2* and *GhLRX19* share high sequence similarity, a VIGS construct targeting a sequence shared by the two homoeologs was used to examine the potential association of this gene pair with plant height.

**Figure 9:**
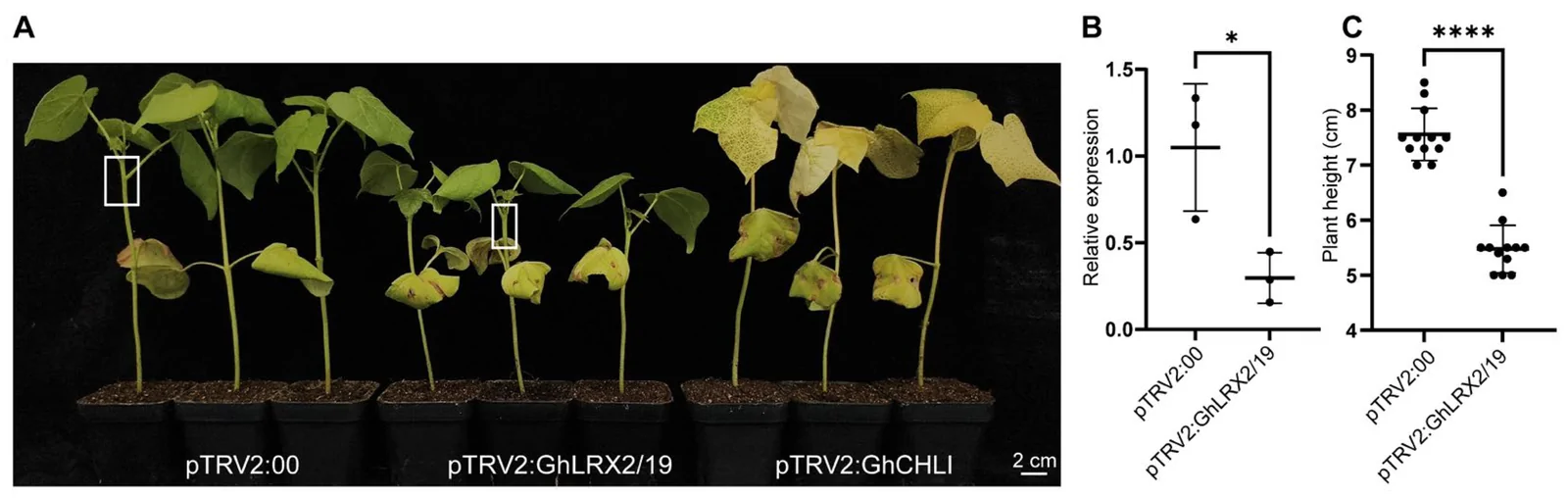
Preliminary investigation of *GhLRX2*/*GhLRX19* using VIGS. (**A**) Phenotypes of pTRV2:00, pTRV2:GhLRX2/19, and pTRV2:GhCHLI plants at 20 days post-inoculation. pTRV2:00 and pTRV2:GhCHLI served as the empty-vector and positive controls, respectively. White boxes indicate the stem regions sampled for anatomical observation. Scale bar = 2 cm. (**B**) Combined relative transcript abundance detected for *GhLRX2*/*GhLRX19* in stem tissues by qRT-PCR. Relative transcript abundance was calculated using the 2^−ΔΔCt^ method, with the mean ΔCt of the three control biological replicates used as the calibrator. Individual points represent three independent biological replicates per group, and data are presented as the mean ± SD. Statistical comparison was performed on biological-replicate ΔCt values using a two-tailed Welch’s *t*-test (*p* = 0.0304). (**C**) Plant height at 20 days post-inoculation, measured from the cotyledonary node to the shoot apical meristem. Points represent 12 individual plants per group, and data are presented as the mean ± SD. Groups were compared using a two-tailed Welch’s *t*-test on individual plant-height measurements (*p* = 1.53 × 10^−10^). Asterisks indicate statistical significance (* *p* < 0.05, **** *p* < 0.0001).

pTRV2:GhCHLI was used as a positive control to assess the effectiveness of the VIGS system. Approximately 15 days after inoculation, the true leaves of the positive-control plants exhibited an obvious bleaching phenotype (Figure 9A; Supplementary Figure S1), indicating that the VIGS system was effective under the experimental conditions. At 20 days post-inoculation, stem tissues were collected from plants treated with the empty-vector control (pTRV2:00) and pTRV2:GhLRX2/19 for qRT-PCR analysis. Compared with the empty-vector control, pTRV2:GhLRX2/19-treated plants showed lower combined transcript abundance detected for *GhLRX2*/*GhLRX19* (Figure 9B). The pTRV2:GhLRX2/19-treated plants also exhibited lower plant height than the empty-vector controls (Figure 9C).

Representative longitudinal stem sections from one pTRV2:00 control plant and one pTRV2:GhLRX2/19-treated plant were additionally examined for descriptive purposes. The corresponding anatomical observations were not subjected to inferential statistical analysis (Supplementary Figure S3). The qRT-PCR and plant-height results provide preliminary evidence for an association between VIGS treatment targeting *GhLRX2*/*GhLRX19* and reduced plant height under the conditions examined.

## 3. Discussion

Plant cell walls are extracellular structures that provide mechanical support while retaining dynamic plasticity. Their compositional reorganization and changes in mechanical properties can directly affect cell elongation, organ development, and environmental adaptation [34,35]. Although the functions of LRX family proteins in vegetative growth, reproductive development, and stress responses have been extensively investigated in *Arabidopsis thaliana*, the patterns of genomic expansion, homoeologous gene retention, and functional differentiation of this family in the allotetraploid crop upland cotton remain poorly understood.

In the present study, 36 nonredundant GhLRX loci were identified from the TM-1 reference genome, compared with 29 reported previously using the same genome assembly [33]. A direct comparison identified 27 shared loci, nine loci included only in the present dataset, and two loci reported only in the previous study. The locus-by-locus correspondence between the two datasets was examined (Table S12), with sequence- and domain-based evidence for candidate retention or exclusion summarized separately (Tables S15 and S16). Across the three cotton species, 75 of the 79 analyzed protein models were predicted to contain Sec/SPI-type signal peptides. The remaining four proteins, GhLRX9, GhLRX15, GhLRX20, and GhLRX36, lacked predicted Sec/SPI signal peptides, and their subcellular localization requires further validation. Differences in LRX family size among species may reflect genome ploidy and family expansion, as well as differences in genome annotation and identification methods [36,37,38]. Notably, 34 of the 36 GhLRX loci were classified as WGD/segmental duplicates. Among the 50 collinear gene pairs involving the identified GhLRX genes, 34 connected the At and Dt subgenomes. Within the identified GhLRX family, 15 At–Dt homoeologous gene pairs were identified. Although duplicated homoeologs often retain a certain degree of functional redundancy, they may also diverge in sequence structure and transcriptional regulation under long-term evolutionary pressures [39,40]. This possibility may also be relevant to the expression profiles of the GhLRX homoeologs identified in this study across different tissues and under different stresses.

Based on the maximum-likelihood phylogenetic tree and with reference to the *Arabidopsis LRX members*, the analyzed proteins were grouped into four classes (Class I–IV) for descriptive purposes. Class I included AtLRX1–5 and AtLRX7, which are primarily associated with vegetative growth, whereas Class II included the pollen-associated proteins AtLRX8–11 [26,27,31,32]. Several Class II genes, including *GhLRX15*, *GhLRX16*, and *GhLRX34*, showed relatively higher transcript abundance in anthers and may therefore warrant further investigation in reproductive tissues.

Among the *Arabidopsis* members of Class I, *AtLRX1* and *AtLRX2* exhibit functional redundancy in root-hair morphogenesis, while the loss of multiple vegetative LRX genes affects cell-wall composition and plant growth [22,30,41]. Some cotton members of this class showed relatively high transcript abundance in ovules, fibers, or stems. In particular, the homoeologous genes *GhLRX2* and *GhLRX19* exhibited relatively high transcript abundance in stems and 20-DPA fibers compared with most other sampled tissues and developmental stages. Their phylogenetic placement and expression profiles provided a basis for selecting this homoeologous pair for preliminary investigation of their potential involvement in cotton growth.

Phytohormones play important roles in plant growth, development, and responses to environmental stresses. ABA participates in drought and other abiotic stress responses [42,43,44], while jasmonate signaling is involved in defense, reproductive development, and cell-wall metabolism [45,46]. Auxin regulates cell-wall loosening and cell expansion and interacts with gibberellins to influence organ growth [47,48]. In this study, promoter sequence analysis predicted putative cis-acting elements annotated as related to ABA, MeJA, auxin, gibberellin, salicylic acid, and low-temperature responses in LRX genes from three cotton species. Among the hormone-related categories, MeJA-responsive elements occurred in the largest number of promoters, followed by ABA- and gibberellin-responsive elements. This distribution describes the occurrence of predicted motifs rather than their enrichment or functional activity. Separately, public transcriptome data revealed variation in GhLRX transcript abundance across tissues, developmental stages, and treatment conditions. Because these analyses use normalized, replicate-averaged expression values, the observed patterns are exploratory and do not establish statistically supported differential expression or stress induction. Together, the promoter annotations and expression profiles provide a basis for developing hypotheses about cotton LRX gene regulation, but they do not demonstrate that the predicted elements account for the observed expression patterns. Testing these relationships will require functional assays of the candidate cis-acting elements and expression analyses with independent biological replicates under controlled treatments.

It should be noted that the VIGS fragment used in this study targeted a conserved sequence shared by *GhLRX2* and *GhLRX19*, which constitute a pair of At- and Dt-subgenome homoeologs. Therefore, the individual contributions and potential functional redundancy of these two homoeologs could not be determined, and possible off-target effects cannot be completely excluded. Under the conditions examined, VIGS treatment was associated with reduced plant height. However, because node number and internode length were not measured separately, it remains unclear whether the observed change in plant height was associated with changes in node number, internode elongation, or both. In addition, the relatively small scale of the VIGS experiment, the lack of homoeolog-specific quantitative analysis, the absence of experimental validation of qRT-PCR primer efficiency and specificity, and the lack of independent biological replication for the anatomical observations limit further interpretation of the results. Thus, the present data provide only preliminary evidence for an association between VIGS treatment targeting the sequence shared by *GhLRX2* and *GhLRX19* and reduced plant height under the tested conditions. Further gene-specific experiments with adequate biological replication are required to clarify the individual functions and genetic relationship of the two homoeologs and the biological basis of the observed phenotype.

## 4. Materials and Methods

### 4.1. Identification of LRX Family Members in Three Cotton Species

The reference genome sequences, predicted protein sequences, and genome annotation files of upland cotton (*Gossypium hirsutum*), *G. arboreum*, and *G. raimondii* were downloaded from CottonGen (https://www.cottongen.org/; accessed on 3 June 2026) (Table S5) [49], and local BLASTP databases were constructed for the three cotton species using TBtools-II v2.487. Protein sequences of *Arabidopsis thaliana* LRX family members were retrieved from The Arabidopsis Information Resource (TAIR; http://www.arabidopsis.org/; accessed on 24 May 2026) [50].

The AtLRX proteins were first used as queries to search the predicted protein databases of the three cotton species using the BLASTP module implemented in TBtools-II v2.487. Hits with an E-value ≤ 1 × 10^−10^ and sequence identity ≥ 40% were retained as initial candidates [33]. No additional query-coverage threshold was applied. These BLASTP-derived candidates were subsequently screened using the HMMER module implemented in TBtools-II v2.487 with the Pfam LRR-related profile PF08263 [51]. The HMMER search was performed using the default settings in TBtools-II v2.487, and no additional manually specified domain-level E-value or coverage cutoff was applied. The corresponding HMM search results are provided (Supplementary File S1). Candidates recovered by the PF08263-based screening were further examined using InterPro (https://www.ebi.ac.uk/interpro/; accessed on 7 October 2026) [52] and SMART (http://smart.embl.de/; accessed on 29 September 2026) [53] to evaluate LRR-related features and overall protein architecture. Because PF08263 is an LRR-related profile rather than an LRX-specific signature, it was used only as an initial screening step.

To improve candidate recovery, the cotton LRX proteins retained after first-round validation were used as queries in a second-round BLASTP search against the predicted protein database of the corresponding cotton species using TBtools-II v2.487. The same BLASTP thresholds were applied. This second-round search was intended to recover additional LRX-like proteins that might have been missed in the initial AtLRX-based search and was not treated as a reciprocal best-hit analysis or as evidence of one-to-one orthology. All proteins meeting the BLASTP criteria in the second round were subjected directly to InterPro and SMART analysis without an additional PF08263-based filtering step. Complete InterProScan results for all second-round candidate proteins are provided (Supplementary File S2). The numbers of candidates recovered at different stages of the identification workflow are summarized (Table S13), and detailed stage-specific identification, retention, and exclusion records are provided (Table S14).

Final family assignment was based on combined evidence from sequence similarity, LRX-family-level annotations, LRR-related features, downstream Cys-rich regions, C-terminal sequence characteristics, and overall protein architecture. Explicit PR01217/PR01218 extensin-related signatures were recorded when detected, but their absence alone was not considered sufficient evidence for exclusion because the C-terminal extensin-like region can vary substantially among LRX proteins. Candidates with short or divergent C-terminal regions were retained when the combined LRR, downstream Cys-rich, C-terminal, and overall architectural evidence supported LRX classification. In contrast, candidates supported mainly by LRR-related similarity but lacking sufficient downstream LRX-like sequence evidence were excluded, as were proteins with architectures inconsistent with LRX proteins, particularly receptor-like kinases. Protein length alone was not used as an exclusion criterion. The identification evidence and final classification of all second-round candidates are summarized (Table S15), whereas detailed sequence- and domain-level evidence supporting the classification of the final retained LRX proteins is provided (Table S16).

For loci with multiple annotated transcript isoforms, all corresponding predicted protein sequences were retained for conserved-motif analysis, and the corresponding transcript annotations were used for exon–intron structure analysis. For phylogenetic analysis, one representative protein sequence was selected per locus; for loci with multiple isoforms, the isoform designated “.1” was used as the representative, namely GrLRX1.1, GrLRX11.1, and GrLRX14.1. Gene-family size, duplication, and expansion were assessed at the gene-locus level. LRX loci were named according to chromosomal position, and alternative transcript isoforms and their corresponding protein isoforms from the same locus were distinguished using decimal suffixes assigned in this study (e.g., GrLRX1.1–GrLRX1.4 for alternative isoforms of the *GrLRX1* locus). The coding sequences (CDSs) of all retained transcript isoforms and their corresponding predicted amino acid sequences are provided (Supplementary Files S3 and S4, respectively).

### 4.2. Physicochemical Property Analysis of LRX Proteins

The amino acid lengths, molecular weights, theoretical isoelectric points, and other physicochemical properties of the LRX proteins were predicted using ExPASy ProtParam (http://web.expasy.org/protparam/; accessed on 1 October 2026) [54]. Chromosomal positions, coding sequence lengths, and gene structure information were obtained from the corresponding reference genome annotation files. SignalP 6.0 (https://services.healthtech.dtu.dk/services/SignalP-6.0/; accessed on 1 October 2026) was used to predict N-terminal Sec/SPI-type signal peptides and to assess the potential classical secretory characteristics of candidate LRX proteins [55]. Because alternative splicing may alter the N-terminal region of a protein and consequently its signal peptide features, SignalP 6.0 was used to predict N-terminal Sec/SPI-type signal peptides separately for all 79 retained predicted protein sequences representing 74 LRX loci, with the organism group set to Eukarya. Predictions were retained for each annotated protein model, including models sharing identical N-terminal sequences.

### 4.3. Phylogenetic, Conserved Motif, and Gene Structure Analyses of the LRX Gene Family

Multiple sequence alignment of LRX protein sequences from upland cotton (*Gossypium hirsutum*), *G. arboreum*, *G. raimondii*, and *Arabidopsis thaliana* was performed using the ClustalW algorithm implemented in MEGA 12 (Supplementary File S6). The best-fitting amino acid substitution model was evaluated using the Find Best Protein Models (ML) function in MEGA 12. WAG + G + I was selected as the best-fitting model based on the lowest Bayesian information criterion (BIC); the corresponding model-selection output, including BIC, corrected Akaike information criterion (AICc), log-likelihood (lnL), and estimated model parameters, is provided as supplementary data (Supplementary File S7). Based on the resulting alignment, a maximum-likelihood (ML) phylogenetic tree was constructed in MEGA 12 using the WAG + G + I model with five discrete gamma categories. Branch support was evaluated using the adaptive bootstrap procedure with a standard-error (SE) stopping threshold of 5%, under which the number of bootstrap replicates was determined adaptively according to the specified SE criterion. Gaps and missing data were handled by partial deletion with a site-coverage cutoff of 95%. The ML tree was inferred using the fast subtree-pruning-regrafting (SPR) heuristic method at level 3, with the initial tree generated automatically (Supplementary File S8) [56]. For descriptive purposes, the resulting phylogeny was divided into four groups (Class I–IV) based on tree topology and the positions of the *Arabidopsis* LRX proteins; these classes were treated as descriptive phylogenetic groupings rather than formally established subfamilies. The phylogenetic tree was edited and visualized using iTOL (https://itol.embl.de/; accessed on 13 September 2026) [57].

Conserved motifs were predicted in all 79 LRX protein sequences representing 74 loci using the MEME online tool (https://meme-suite.org/meme/tools/meme; accessed on 29 September 2026), with the maximum number of motifs set to 10. The exon–intron structures of the corresponding 79 annotated LRX transcript models were obtained from the GFF annotation files. Transcript structures and conserved protein motif distributions were visualized using TBtools-II [58,59].

### 4.4. Cis-Acting Element Analysis of Cotton LRX Genes

For each of the 74 LRX loci, a 2000-bp sequence upstream of the translation start site of the representative transcript model was extracted using TBtools-II and defined as the promoter region, yielding one promoter sequence per locus (Supplementary File S5). For loci with multiple annotated transcript models, the representative transcript models (*GrLRX1.1*, *GrLRX11.1*, and *GrLRX14.1*) were used for promoter extraction. Cis-acting elements were predicted using PlantCARE (http://bioinformatics.psb.ugent.be/webtools/plantcare/html/; accessed on 29 September 2026) [60]. Regulatory elements associated with light responses, phytohormone responses, and abiotic stress responses were prioritized, and the prediction results were visualized using TBtools-II.

### 4.5. Chromosomal Localization and Collinearity Analysis of the GhLRX Gene Family

The chromosomal positions of *GhLRX* genes were extracted from the TM-1 annotation file, and chromosomal localization maps were generated using TBtools-II. Intraspecific collinearity analysis was performed using the One Step MCScanX module in TBtools-II v2.487, and collinearity maps were generated using the Advanced Circos module [61]. *Ka/Ks* values for collinear gene pairs involving identified GhLRX loci were calculated using the Simple *Ka/Ks* Calculator in TBtools-II [59].

### 4.6. Interspecific Collinearity Analysis

Reference genome sequences in FASTA format and annotation files in GFF format for *Gossypium arboreum* and *Gossypium raimondii* were downloaded from CottonGen (https://www.cottongen.org/; accessed on 3 June 2026) [49]. The Genome Length Filter module in TBtools-II v2.487 was used to organize chromosome-length information and genome annotations. Interspecific collinearity analysis was performed using the One Step MCScanX module in TBtools-II. Collinearity relationships between *G. arboreum* and *G. hirsutum*, and between *G. hirsutum* and *G. raimondii*, were constructed and visualized using the Multiple Synteny Plot module in TBtools-II [62].

### 4.7. Expression Analysis of GhLRX Genes Across Tissues, Developmental Stages, and Abiotic Stress Conditions

To investigate the expression patterns of *GhLRX* genes across different tissues, developmental stages, and stress conditions, transcriptomic expression data for upland cotton accession TM-1 were downloaded from the Zhejiang University Cotton Database (http://cotton.zju.edu.cn/; accessed on 30 September 2026). The dataset comprised samples collected from different tissues and developmental stages, as well as samples subjected to different stress treatments, with two or three biological replicates per sample. The expression data from the biological replicates had already been normalized and averaged in the database.

The expression values of *GhLRX* genes were extracted according to the sample annotation information provided by the database and used to construct separate expression matrices for tissues/developmental stages and stress treatments (Tables S7 and S8). Heatmaps were subsequently generated using TBtools-II v2.487. To highlight the relative expression variation in each *GhLRX* gene across tissues or developmental stages and among stress-treatment conditions, row-wise scaling (Row Scale) was applied separately to the expression matrix of each heatmap. For each gene in each heatmap, the expression values were converted to *Z*-scores based on the mean and standard deviation of that gene across all samples included in the corresponding heatmap. After row-wise scaling, positive values indicate that the expression level of a gene in a given sample was higher than its mean expression level across the samples included in that heatmap, whereas negative values indicate expression below its mean level. This transformation was used solely to visualize the relative expression pattern of each gene across samples; no column-wise scaling was performed. Because the tissue/developmental-stage and stress-treatment heatmaps were row-scaled independently, the resulting *Z*-scores are comparable only within each respective heatmap and should not be used to compare absolute expression levels between the two heatmaps.

### 4.8. VIGS-Mediated Targeting of GhLRX2 and GhLRX19 in Upland Cotton

Based on the expression patterns revealed by publicly available transcriptomic data, we hypothesized that the homoeologous genes *GhLRX2* and *GhLRX19* may be associated with cotton stem development. Therefore, the two genes were selected for preliminary investigation using virus-induced gene silencing (VIGS). CCRI24 was selected as the recipient cultivar for VIGS. A 300-bp VIGS fragment was designed based on the CDS sequence of *GhLRX19*, commercially synthesized, and subsequently cloned into the pTRV2 vector to generate the recombinant vector pTRV2:GhLRX2/19. The plasmids pTRV1, pTRV2:00, pTRV2:GhLRX2/19, and pTRV2:GhCHLI were then individually introduced into competent *Agrobacterium tumefaciens* GV3101 cells.

Single positive colonies were cultured in LB liquid medium containing kanamycin and rifampicin at 28 °C and 200 rpm until the culture reached an OD_600_ of 0.8–1.0. The bacterial cells were collected and resuspended in infiltration buffer containing 10 mM MgCl_2_, 10 mM MES, and 200 μM acetosyringone (pH 5.6), followed by incubation at room temperature for 3–4 h. The Agrobacterium suspension carrying pTRV1 was separately mixed with suspensions carrying pTRV2:00, pTRV2:GhLRX2/19, or pTRV2:GhCHLI at a 1:1 volume ratio [63].

When the cotyledons had fully expanded, the mixed bacterial suspensions were infiltrated into the abaxial surfaces of the cotyledons using a 1-mL needleless syringe. After infiltration, the plants were kept in the dark at 22–23 °C for 24 h and then transferred to a 16-h-light/8-h-dark photoperiod. Plants inoculated with pTRV2:GhCHLI and pTRV2:00 were used as the positive and empty-vector controls, respectively [64]. A preliminary VIGS experiment was first conducted to qualitatively assess whether targeting *GhLRX2*/*GhLRX19* was accompanied by an observable plant-height phenotype. Individual plant heights were not systematically measured in this preliminary experiment. A second independent VIGS experiment was subsequently conducted under standardized growth and handling conditions. In this experiment, plant height was quantitatively measured in 12 independent plants per group at 20 days post-inoculation, and these data were used for statistical analysis.

### 4.9. RNA Extraction and Quantitative Real-Time PCR Analysis

At 20 days post-inoculation, plant height was measured in 12 individual plants per group as the distance from the cotyledonary node to the shoot apical meristem (Table S9). Stem tissues were then collected from three individual plants in the control group and three individual plants in the VIGS-targeted group for RNA extraction. Each individual plant was treated as one independent biological replicate, and RNA was extracted separately from each plant using the EASYspin Plus Plant RNA Kit (RN38 series; Aidlab Biotechnologies Co., Ltd., Beijing, China). First-strand cDNA was synthesized from the extracted RNA using HiScript IV RT SuperMix (Vazyme, Nanjing, China), according to the manufacturer’s instructions. Quantitative real-time PCR was performed on a LightCycler 480 II real-time PCR system (Roche Diagnostics International Ltd., Rotkreuz, Switzerland) using ChamQ Universal SYBR qPCR Master Mix (Vazyme, Nanjing, China; Cat. No. Q711-02). *GhPP2A1* was used as the reference gene, and the primers used in this study are listed (Table S6). Three independent biological replicates were analyzed for each group, and four technical replicates were initially performed for each target- and reference-gene assay within each biological replicate. The target and reference genes were amplified in separate reaction wells. The qRT-PCR conditions were as follows: initial denaturation at 95 °C for 5 min, followed by 40 cycles of 95 °C for 10 s, 60 °C for 15 s, and 72 °C for 20 s. For each biological replicate, valid technical-replicate Ct values were averaged separately for the target- and reference-gene assays. ΔCt was calculated by subtracting the mean *GhPP2A1* Ct value from the mean target-gene Ct value. The mean ΔCt of the three control biological replicates was used as the common calibrator for calculation of ΔΔCt, and relative transcript abundance was calculated using the 2^−ΔΔCt^ method [65]. The raw Ct values are provided in the Supplementary Materials (Table S10). Amplification efficiencies were not experimentally determined, and independent experimental validation of amplicon specificity was not performed.

### 4.10. Paraffin Sectioning and Cell Length Measurement

For descriptive anatomical comparison, stem segments were collected from the same developmental position near the shoot apical meristem of control and pTRV2:GhLRX2/19-treated plants at 20 days post-inoculation. The sampling positions are indicated in Figure 9A. The pTRV2:GhLRX2/19-treated plant used for paraffin-section analysis was examined by qRT-PCR, and the combined transcript abundance detected for *GhLRX2* and *GhLRX19* was lower than that in the control plant. The collected stem segments were fixed and processed for paraffin embedding according to the published protocol [66]. Samples were dehydrated through a graded ethanol series and embedded in paraffin. Longitudinal sections (2 μm thick) were prepared using a Leica RM2245 microtome, deparaffinized, stained with 1% (*w*/*v*) Safranin O for 1 h, differentiated in ethanol, and counterstained with 0.5% (*w*/*v*) Fast Green for 30 s. Following final dehydration and drying, the sections were examined under a light microscope, and images were viewed and exported using CaseViewer version 2.4.0.119028 (Supplementary Figure S2). The longitudinal lengths of cortical parenchyma cells were measured using ImageJ software (version 1.54g) [67]. Cells with clearly defined boundaries and cell axes approximately parallel to the longitudinal axis of the stem were selected for measurement. One plant from each group was used for paraffin-section analysis, and 20 cortical parenchyma cells were measured from each plant. These measurements were used solely for within-plant descriptive comparison, and no inferential statistical test was performed on the cell-length data (Supplementary Figure S3).

### 4.11. Statistical Analysis

Statistical analyses were performed using GraphPad Prism version 10.6.0. For plant-height analysis, 12 individual plants per group were treated as independent biological units. For qRT-PCR analysis, three independently sampled plants per group were treated as independent biological replicates, with four technical replicates initially performed for each target- and reference-gene assay. The target- and reference-gene assays were performed in separate reaction wells, and their technical replicates were not treated as paired observations. In biological replicate 3 of the pTRV2:00 group, one reference-gene Ct value (27.57) was considered anomalous because it markedly deviated from the other three reference-gene technical replicates. Similarly, in biological replicate 1 of the pTRV2:GhLRX2/19 group, one target-gene Ct value (11.7) markedly deviated from the other three target-gene technical replicates. No predefined numerical Ct cutoff had been established before the experiment. These two anomalous Ct values were excluded from the calculations, whereas the corresponding valid measurements from the separate assays (Ct = 20.57 and 18.31, respectively) were retained. Valid technical-replicate Ct values were averaged separately for the target- and reference-gene assays within each biological replicate before calculation of ΔCt. Statistical comparison was performed on the biological-replicate ΔCt values using a two-tailed Welch’s *t*-test. Technical replicates were not treated as independent observations. Given the small number of biological replicates in the qRT-PCR analysis (*n* = 3 per group), formal assessment of distributional assumptions was considered insufficiently informative and was not used as the basis for statistical inference.

Plant-height distributions were assessed using normal Q–Q plots and the Shapiro–Wilk test. Differences between the pTRV2:00 and pTRV2:GhLRX2/19 groups were evaluated using a two-tailed Welch’s *t*-test based on the 12 individual plant-height measurements per group. Data are presented as the mean ± SD, and *p* < 0.05 was considered statistically significant. Exact *p*-values are reported in the corresponding figure legend. Anatomical cell-length measurements were used solely for within-sample descriptive comparison and were not subjected to inferential statistical analysis.

## 5. Conclusions

In this study, we conducted a comparative genome-wide characterization of the LRX gene family in *Gossypium hirsutum*, *G. arboreum*, and *G. raimondii*, identifying 74 LRX gene loci across the three cotton species. Comparative analyses revealed conserved and divergent features in their phylogenetic relationships, gene structures, chromosomal distributions, collinearity patterns, promoter cis-acting elements, and expression profiles. The homoeologous genes *GhLRX2* and *GhLRX19* were selected for preliminary functional investigation based on their expression patterns. VIGS treatment targeting a sequence shared by *GhLRX2* and *GhLRX19* was associated with lower combined transcript abundance detected for the two genes and reduced plant height. These findings provide an updated resource for the cotton LRX gene family and preliminary evidence for an association between VIGS treatment targeting *GhLRX2*/*GhLRX19* and reduced plant height, while the individual responses and functions of the two homoeologs remain to be determined.

## Data Availability

The data supporting the findings of this study are provided in the article and its Supplementary Materials. Candidate counts, locus comparisons, and identification records are provided (Tables S12–S16); HMM-based search results are provided (Supplementary File S1). Complete InterProScan output is provided (Supplementary File S2), with candidate-level sequence- and domain-based summaries provided separately (Tables S15 and S16). Coding sequences, protein sequences, promoter sequences, the multiple-sequence alignment, model-selection results, and the maximum-likelihood tree are provided (Supplementary Files S3–S8, respectively). The expression matrices (Tables S7 and S8) were obtained from the Zhejiang University Cotton Database and contain normalized values averaged across biological replicates provided by the database; they do not represent raw RNA-seq read counts. Individual plant-height measurements and raw qRT-PCR Ct values are provided (Tables S9 and S10, respectively). Original uncropped phenotype photographs and microscopy images are provided (Supplementary Figures S1 and S2), and the descriptive anatomical observations are provided (Supplementary Figure S3). SMART was used as a complementary tool for domain evaluation, but complete SMART output files were not generated and are therefore not provided. Further inquiries should be directed to the corresponding authors.

## Supplementary Materials

The following supporting information can be downloaded at: https://www.mdpi.com/article/10.3390/ijms27209119/s1.

## Abbreviations

The following abbreviations are used in this manuscript:

LRX	leucine-rich repeat extensin
LRR	leucine-rich repeat
ABA	abscisic acid
MeJA	methyl jasmonate
GA	gibberellin
SA	salicylic acid
JA	jasmonic acid
ROS	reactive oxygen species
WGD	whole-genome duplication
Ka/Ks	ratio of nonsynonymous substitution rate to synonymous substitution rate
DPA	days post-anthesis
VIGS	virus-induced gene silencing
