# Supplementary material for: Genome-Wide Characterization of the LRX Gene Family in Three Cotton Species and Preliminary Evidence Linking GhLRX2/GhLRX19 to Plant Height in Upland Cotton
Source: Int J Mol Sci. 2026 Oct 14;27(20):9119. doi: 10.3390/ijms27209119 (PMC13705520; doi:10.3390/ijms27209119)
Supplement: Supplementary file 1 [file ijms-27-09119-s001.zip › Supplementary Figure S3. Descriptive anatomical observations of stem longitudinal sections following VIGS treatment.pdf]

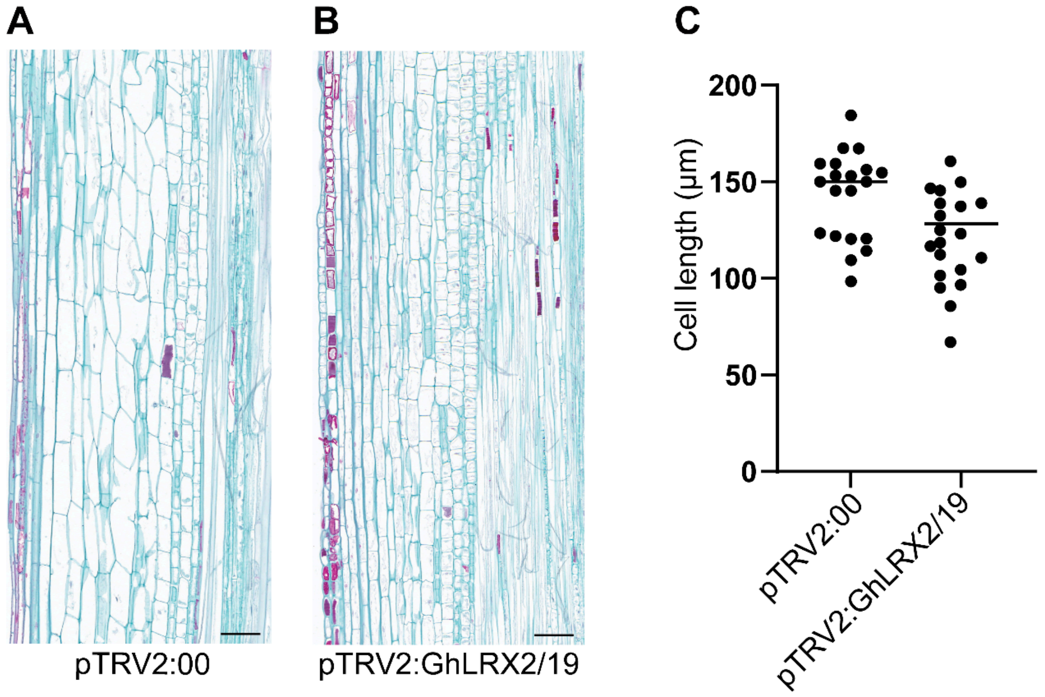

Supplementary Figure S3. Descriptive anatomical observations of stem tissues from pTRV2:00 and pTRV2:GhLRX2/19-treated cotton plants. (**A**) Representative longitudinal paraffin section of stem tissue from one pTRV2:00 control plant. Scale bar = 100 µm. (**B**) Representative longitudinal paraffin section of stem tissue from one pTRV2:GhLRX2/19-treated plant sampled from the same developmental position as the control plant. Scale bar = 100 µm. (**C**) Within-sample distribution of cortical parenchyma cell lengths measured in the sections shown in (**A**,**B**). Twenty cells were measured from each examined plant, and individual points represent individual cell measurements; horizontal lines indicate the mean values. Because only one plant per group was examined, these measurements represent within-plant observations rather than independent biological replicates and are presented for descriptive purposes only. No inferential statistical analysis was performed.
